# Ultra-superovulation for the CRISPR-Cas9-mediated production of gene-knockout, single-amino-acid-substituted, and floxed mice

**DOI:** 10.1242/bio.019349

**Published:** 2016-07-07

**Authors:** Yoshiko Nakagawa, Tetsushi Sakuma, Norihisa Nishimichi, Yasuyuki Yokosaki, Noriyuki Yanaka, Toru Takeo, Naomi Nakagata, Takashi Yamamoto

**Affiliations:** 1 Center for Animal Resources and Development, Kumamoto University, 2-2-1 Honjo, Chuo-ku, Kumamoto 860-0811, Japan; 2 Department of Mathematical and Life Sciences, Graduate School of Science, Hiroshima University, 1-3-1 Kagamiyama, Higashi-Hiroshima, Hiroshima 739-8526, Japan; 3 Cell-Matrix Frontier Laboratory, Health Administration Center, Hiroshima University, 1-2-3 Kasumi, Minamiku, Hiroshima 734-8551, Japan; 4 Clinical Genetics, Hiroshima University Hospital, 1-2-3 Kasumi, Minamiku, Hiroshima 734-8551, Japan; 5 Department of Molecular and Applied Bioscience, Graduate School of Biosphere Science, Hiroshima University, 1-4-4 Kagamiyama, Higashi-Hiroshima, Hiroshima 739-8528, Japan

**Keywords:** Knockout, Floxed, CRISPR-Cas9, Zygote, Ultra-superovulation, Inhibin antiserum and equine chorionic gonadotropin (IASe)

## Abstract

Current advances in producing genetically modified mice using genome-editing technologies have indicated the need for improvement of limiting factors including zygote collection for microinjection and their cryopreservation. Recently, we developed a novel superovulation technique using inhibin antiserum and equine chorionic gonadotropin to promote follicle growth. This method enabled the increased production of fertilized oocytes via *in vitro* fertilization compared with the conventional superovulation method. Here, we verify that the ultra-superovulation technique can be used for the efficient generation of clustered regularly interspaced short palindromic repeats (CRISPR)-CRISPR-associated protein 9 (Cas9)-mediated knockout mice by microinjection of plasmid vector or ribonucleoprotein into zygotes. We also investigated whether single-amino-acid-substituted mice and conditional knockout mice could be generated. Founder mice bearing base substitutions were generated more efficiently by co-microinjection of Cas9 protein, a guide RNA and single-stranded oligodeoxynucleotide (ssODN) than by plasmid microinjection with ssODN. The conditional allele was successfully introduced by the one-step insertion of an ssODN designed to carry an exon flanked by two loxP sequences and homology arms using a double-cut CRISPR-Cas9 strategy. Our study presents a useful method for the CRISPR-Cas9-based generation of genetically modified mice from the viewpoints of animal welfare and work efficiency.

## Abstract (precis)

**Summary:** We demonstrate the production of CRISPR-Cas9-mediated knockout and knock-in mice using a recently developed ultra-superovulation technique to obtain greater numbers of oocytes compared with conventional methods.

## INTRODUCTION

Reproductive engineering techniques are essential for the efficient production and maintenance of genetically modified (GM) mice. Fertilized oocytes generated by mating or *in vitro* fertilization (IVF) are used for microinjection with or without cryopreservation to generate genome-edited mice. We have previously shown that IVF and freeze-thawing techniques efficiently generated GM mice using genome-editing technology (Nakagawa et al., 2014, 2015). The use of freeze-thawed fertilized oocytes allows flexible scheduling by allowing the required number of fertilized oocytes to be utilized at any time.

Use of the superovulation technique using equine chorionic gonadotropin (eCG) and human chorionic gonadotropin (hCG) is important to collect as many oocytes as possible from female mice. To further increase the yield of oocytes, we recently developed a new ultra-superovulation technique using inhibin antiserum (IAS) and eCG (IASe) to promote follicle growth (Takeo and Nakagata, 2015). We have shown that approximately threefold numbers of oocytes could be collected from C57BL/6 female mice at 4 weeks of age by the administration of IASe compared with the conventional administration of IAS or eCG alone. We and other investigators have confirmed that fertilized oocytes created by IVF via the conventional superovulation method were applicable for creating genome-edited mice (Nakagawa et al., 2014, 2015; Li et al., 2014; Miura et al., 2015; Tsuchiya et al., 2015; Kaneko and Mashimo, 2015; Nakao et al., 2016; Sakurai et al., 2016). However, it has not yet been elucidated whether the ultra-superovulation technique can be applied for the generation of genome-edited mice.

Recently, various genome-edited mice have been quickly generated using the microinjection technique and the clustered regularly interspaced short palindromic repeats (CRISPR)-CRISPR-associated protein 9 (Cas9) system (Singh et al., 2015). The CRISPR-Cas9 system induces DNA double-strand breaks (DSBs) by forming a complex of Cas9 and single guide RNA (gRNA) that targets a desired genomic locus. The DSBs are mainly repaired by error-prone non-homologous end-joining, which introduces random insertions and deletions of nucleotides around the target site, resulting in targeted gene disruption. Alternatively, DSBs can also be repaired using homology-directed repair (HDR) by co-injection of donor DNA harboring homology arms. Using genome editing technology and this repair system, precisely defined modifications can be efficiently introduced into the targeted locus. The CRISPR-Cas9 system and single-stranded oligodeoxynucleotide (ssODN) carrying objective arrangements with small modifications, including point mutations, multiple base substitutions, and insertion of tag sequences such as loxP, have been often used for the generation of genome-edited mice (Wang et al., 2013, 2015; Yang et al., 2013; Mizuno et al., 2014; Inui et al., 2014; Han et al., 2015; Hashimoto and Takemoto, 2015; Qin et al., 2015; Kaneko and Mashimo, 2015).

**Fig. 1.**
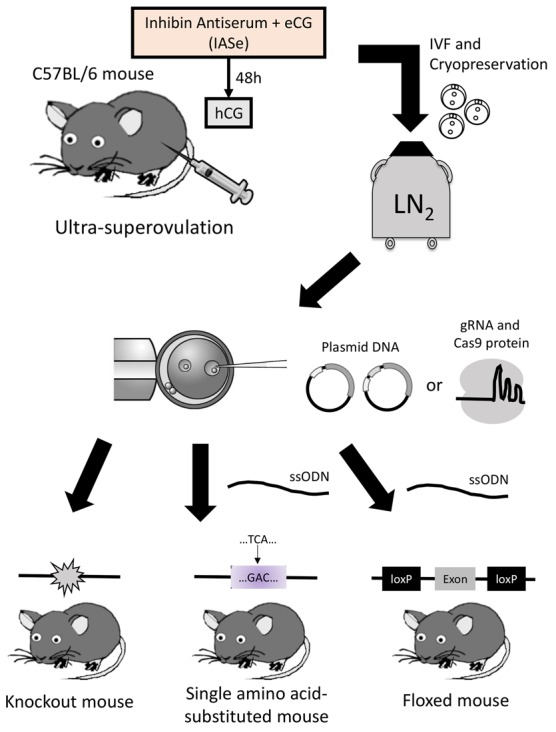
**Schematic overview of the study.** Sexually mature or immature female mice were treated with the ultra-superovulation method. Fertilized oocytes were produced by IVF, and then cryopreserved. After thawing the oocytes, microinjection was performed using the plasmid DNA or Cas9 RNPs with or without ssODNs. Three kinds of genetically modified mice were generated.

Here, we report the CRISPR-Cas9-mediated generation of various GM mice, such as gene-disrupted mice, single-amino-acid-substituted mice, and floxed mice, using zygotes created by IVF via an ultra-superovulation method (Fig. 1). We generated gene-knockout mice by microinjection of an all-in-one CRISPR-Cas9 plasmid vector into zygotes, and subsequently produced the mice bearing three-base substitutions or floxed alleles by using *in vitro* transcribed gRNA and Cas9 protein with ssODN. This study reports an efficient method for creating various genome-edited mice using freeze-thawed zygotes created via ultra-superovulation and IVF.

## RESULTS

### Generation of gene-knockout mice using freeze-thawed fertilized oocytes created by IVF using an ultra-superovulation method

To examine whether freeze-thawed fertilized oocytes created by IVF using an ultra-superovulation method can be applied to the CRISPR-Cas9-mediated generation of knockout mice, the birth and mutation rates of these oocytes injected with CRISPR-Cas9 plasmid vectors were compared with oocytes previously created by IVF using the conventional superovulation method.

**Fig. 2.**
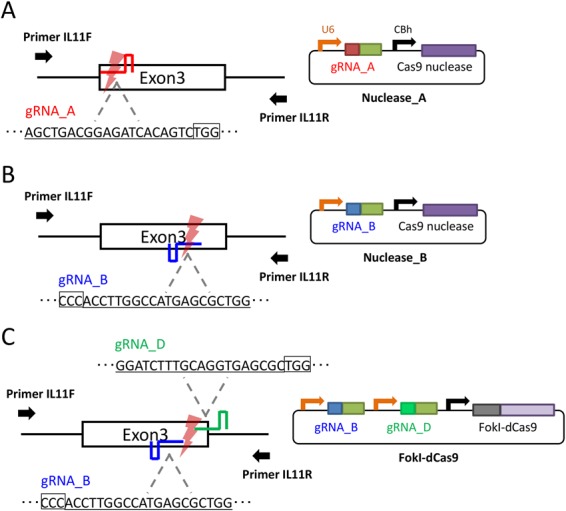
**Generation of knockout mice at the *Il11* locus.** The genomic region around exon 3 of the *Il11* gene was targeted by Cas9 nuclease_A (A), Cas9 nuclease_B (B), or FokI-dCas9 (C) vectors. The target sequence of each gRNA is underlined. The PAM sequence is enclosed in a black box. U6, human U6 promoter; CBh, chicken beta-actin short promoter.

First, we generated fertilized oocytes by IVF using sexually matured C57BL/6 female and male mice. Female mice were treated by the ultra-superovulation method using IASe to promote follicle growth and hCG to induce ovulation. Consistent with our previous report, we collected greater numbers of oocytes compared with the conventional superovulation method (Table S1). The fertilized oocytes were cryopreserved as the stock for microinjection. Next, previously constructed and validated CRISPR-Cas9 nuclease vectors or a FokI-dCas9 vector to target the interleukin-11 (*Il11*) gene (Fig. 2) (Nakagawa et al., 2015) were microinjected into the pronucleus of freeze-thawed fertilized oocytes generated using ultra-superovulated sexually mature mice. Then, the surviving oocytes were transferred to pseudopregnant mice and the resultant pups were obtained. Tail lysates from pups were used for polymerase chain reaction (PCR) to amplify the targeted sequence of *Il11*. Each PCR product was analyzed by direct sequencing. In the previous report, we investigated the birth rate and the mutation rate of zygotes injected with these vectors. Microinjection of Cas9 nuclease vectors resulted in relatively low birth rate and high mutation rate, whereas microinjection of FokI-dCas9 vector resulted in moderate birth and mutation rates (Nakagawa et al., 2015). The birth and mutation rates of zygotes generated using the ultra-superovulation method were similar to those created by the conventional method (Table 1). To examine birth and mutation rates using zygotes from sexually immature female mice, from which greater numbers of fertilized oocytes can be collected compared with mature females, we performed microinjection and transfer using the same method. Although we confirmed that gene-knockout mice could also be created using ultra-superovulated sexually immature female mice, the birth rate was lower than using oocytes from mature females (Table 1). To decrease the toxicity, we subsequently tested microinjection of Cas9 protein and synthesized gRNA_B instead of the plasmid vector (Nuclease_B) into the zygotes generated from immature female mice. This alteration resulted in the successful production of mutant pups at the Nuclease_B locus, although no pups were born by plasmid microinjection, suggesting that the birth rate of genome-edited mice can be improved by replacing CRISPR-Cas9 vectors with Cas9 ribonucleoproteins (RNPs) (Table 1). Collectively, our ultra-superovulation technique was confirmed to be applicable to the production of knockout mice using genome-editing technology.

**Table 1.**
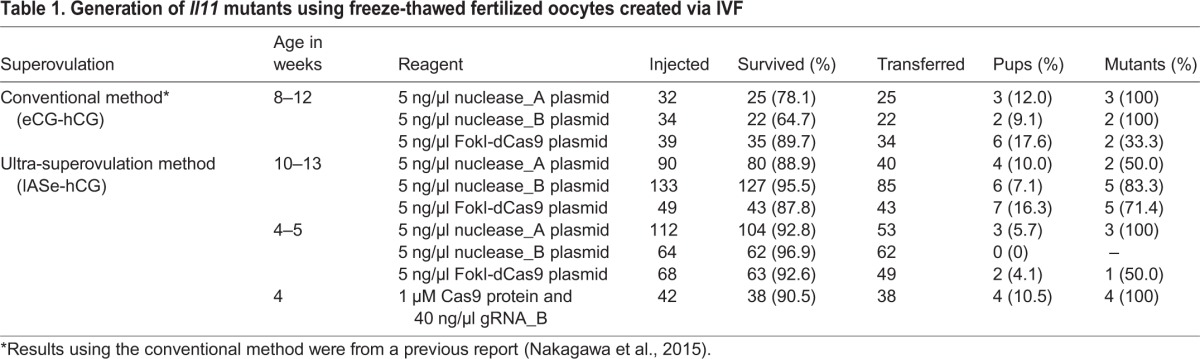
**Generation of *Il11* mutants using freeze-thawed fertilized oocytes created via IVF**

### Generation of single-amino-acid-substituted mice

**Fig. 3.**
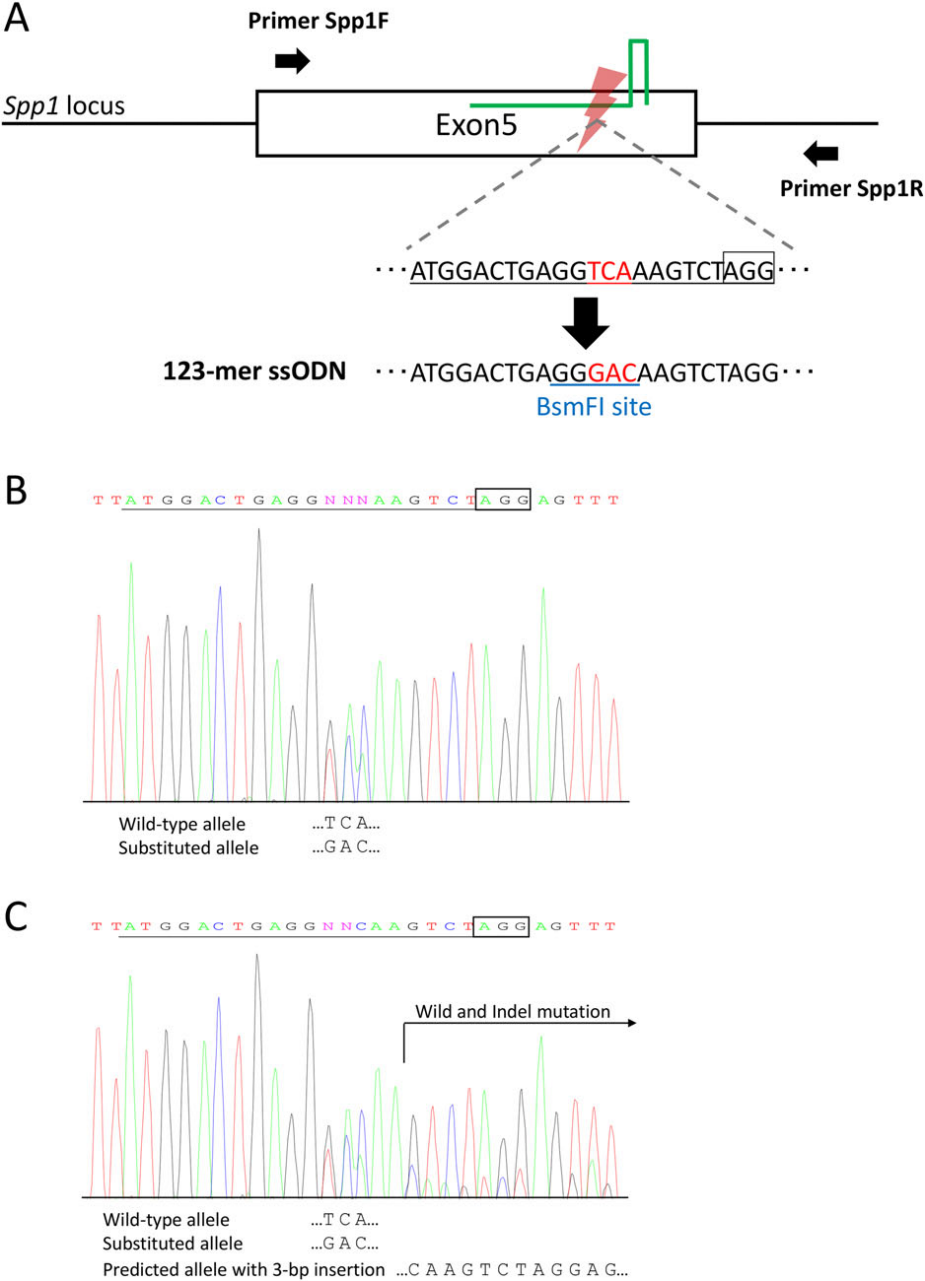
**Generation of single-amino-acid-substituted mice at the *Spp1* locus.** (A) Schematic illustration to generate a three-base substituted allele at the *Spp1* locus. A serine residue in exon 5 was replaced with an aspartic acid (TCA to GAC; red letters). A gRNA was designed to cut in the close vicinity of the serine residue (underlined in black and red). An ssODN was designed to carry the three-base substitution. A black box indicates the PAM sequence. Arrows indicate the primer sets for PCR. Blue underline indicates the recognition site of restriction enzymes in the RFLP analysis. (B,C) The sequencing analyses of *Spp1*-targeted mice. (A) Example of a three-base substitution detected with the wild-type allele. (B) Example of a three-base substitution detected with the wild-type allele and indel mutation. The target sequence of gRNA is indicated by black underline. The PAM sequence is enclosed in a black box.

To investigate further whether gene knock-in mice can be generated using freeze-thawed zygotes created via ultra-superovulation and IVF, as well as the production of gene-knockout mice, we aimed to generate founder mice harboring a three-base substitution in the secreted phosphoprotein 1 (*Spp1*) gene. The three-base substitution in *Spp1* gene was designed to encode thrombin cleavage-incompetent mutant osteopontin protein (Nishimichi et al., 2011). To knock-in these substitutions precisely, we designed a gRNA on the target locus as well as the ssODN carrying the intended substitutions (Fig. 3A).

We initially tested the microinjection of CRISPR-Cas9 nuclease vector and ssODN, followed by single blastocyst assays for the validation of gene knock-in. The surviving zygotes were cultured for 3.5 days, and restriction fragment length polymorphism (RFLP) and direct sequencing analyses were conducted. We confirmed correct three-base substitution without any insertion and deletion (indel) mutations in one blastocyst (Fig. S1, Table S2). To generate the founder mice, we subsequently performed microinjection of CRISPR-Cas9 nuclease vector and ssODN, and then surviving zygotes were transferred to pseudopregnant mice. We used the oocytes collected from immature or mature female mice treated with ultra-superovulation for IVF. The birth rates were low and no pups with the three-base substitution were detected by RFLP analysis using *Bsm*FI (Table 2). Therefore, to improve the birth rate and the knock-in rate, Cas9 RNPs were injected with ssODN into zygotes instead of the CRISPR-Cas9 nuclease vector, similar to the method used for *Il11* targeting. By using Cas9 RNPs, both the birth rate and the knock-in rate were significantly improved (Table 2). The precise knock-in of the three-base substitution was confirmed in three pups by RFLP analysis and direct sequencing of PCR amplicons (Table 2, Fig. 3B). In six pups, the three-base substitution was detected with indel mutations (Table 2, Fig. 3C). Together with the *Il11* results, we confirmed increased birth rate and knockout/knock-in rates by using Cas9 RNPs.

**Table 2.**
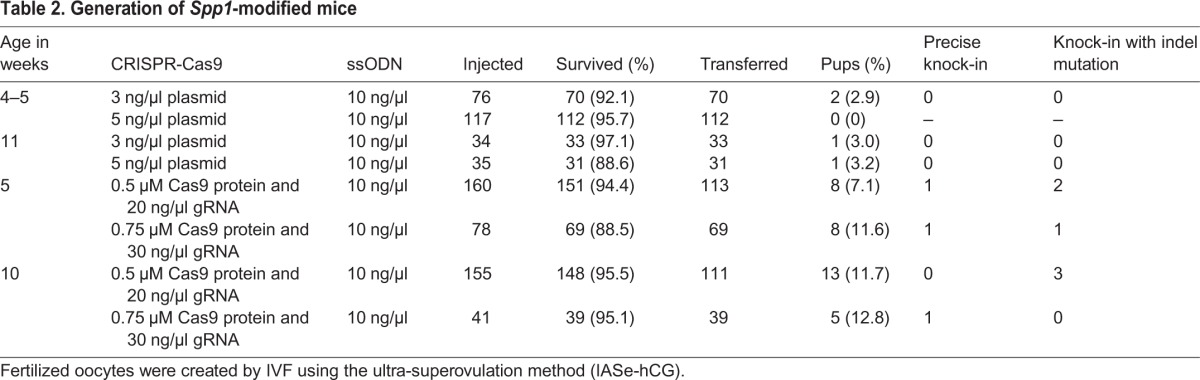
**Generation of *Spp1*-modified mice**

### Design and validation of two loxP insertions

**Fig. 4.**
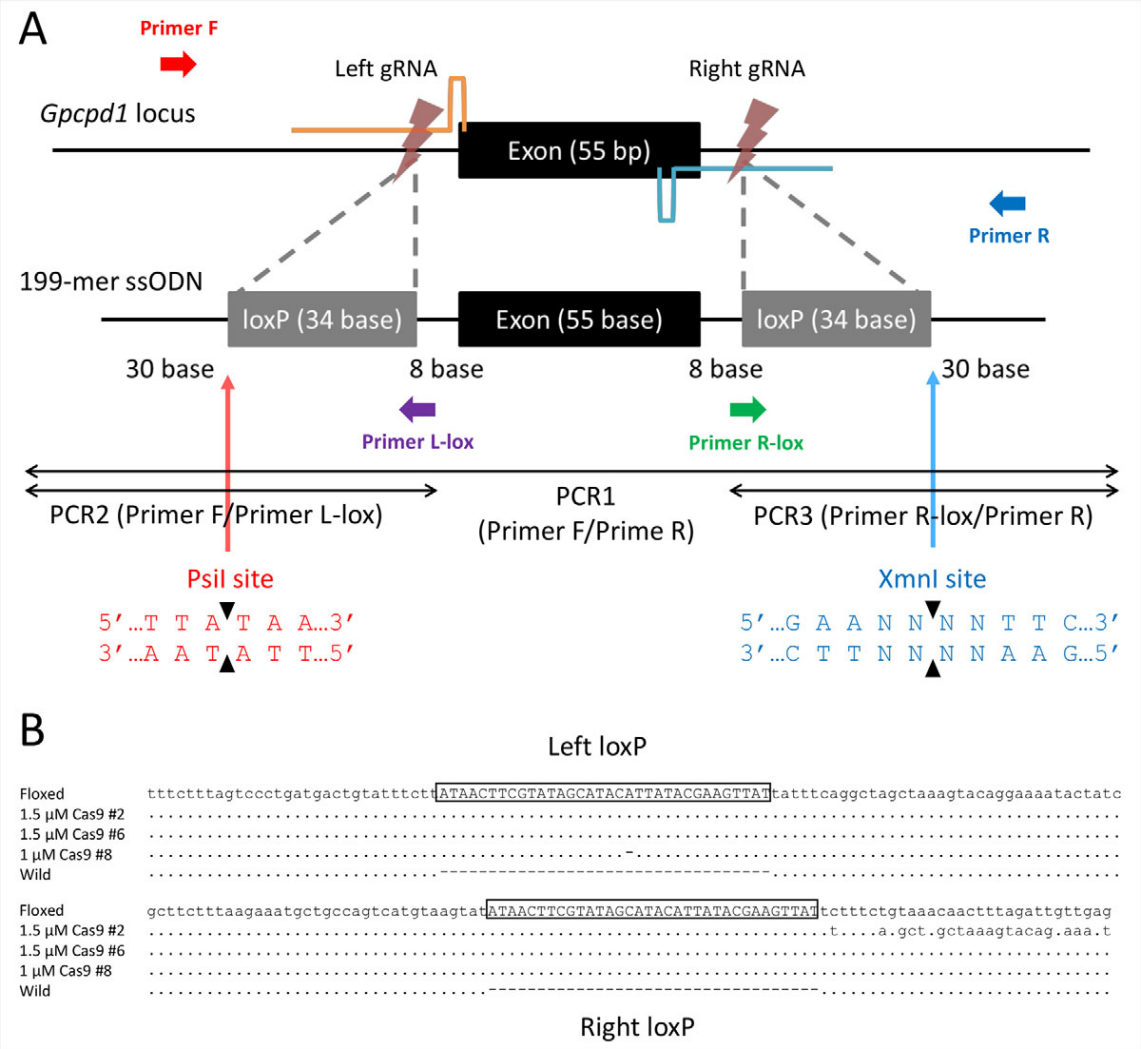
**Generation of floxed mice at the *Gpcpd1* locus.** (A) Schematic illustration to generate a conditional allele at the *Gpcpd1* locus. A 55-bp exon was targeted by two gRNA, which were designed to cut both ends of the exon. A single ssODN was designed to carry the two loxP sequences. Bold arrows indicate primers for PCR. Thin arrows indicate the recognition sites of restriction enzymes for the RFLP analysis. (B) Sequencing analysis of subcloned PCR1 products from three pups that were positive for all analyses. The intended floxed allele is shown at the top (Floxed) with loxP sequences (enclosed in black boxes). The wild-type allele is shown at the bottom. Dots indicate the same bases as the floxed sequence.

We designed a novel strategy for the one-step generation of floxed mice by utilizing double-cut CRISPR-Cas9 and a single ssODN donor. To perform a proof-of-concept examination of this strategy, we constructed an all-in-one CRISPR-Cas9 vector expressing two gRNAs to target the both regions outside a 55-bp exon in glycerophosphocholine phosphodiesterase 1 (*Gpcpd1*), also known as *Gde5* (Okazaki et al., 2010) (Fig. 4A). The ssODN was designed to carry the 55-bp exon flanked by the loxP sequences and 30-bp homology arms.

To confirm the cleavage activity of the all-in-one CRISPR-Cas9 vector and the integration rate of ssODN, the vector and ssODN were microinjected into the pronucleus of zygotes. The resultant 115 surviving oocytes were cultured for 3.5 days. We observed 33 blastocysts, and each blastocyst was used for single blastocyst assays. To roughly genotype the blastocysts, three kinds of PCR were performed (Fig. 4A): PCR1 to amplify the whole targeted locus, PCR2 to detect the integration of the left loxP, and PCR3 to detect the integration of the right loxP. Additionally, we performed RFLP analysis to confirm the integration of the left and right loxP sequences in the PCR1 products using *Psi*I and *Xmn*I, respectively (Fig. 4A). The result of PCR1 suggested that one blastocyst (#19) had a floxed allele (Fig. S2A). Consistent with this result, blastocyst #19 was also positive in PCR2 and *Psi*I-RFLP (Figs S2B, S3A). In PCR3 and *Xmn*I-RFLP, however, four (#11, #14, #25, and #27) and six (#1, #11, #14, #19, #21, and #27) blastocysts were estimated to be positive, respectively (Fig. S2C, Fig. S3B). These discordances might be caused by various patterns of partial integrations, such as the integration of only one loxP sequence and/or integration with indel mutations, as well as the difficulty of performing single blastocyst assays because of the small amount of template genome available. To ensure the exact genotype, the six PCR1 products from blastocysts #1, #11, #14, #19, #21, and #27, were analyzed by direct sequencing. Consequently, we confirmed the nearly correct integration of ssODN in one blastocyst (#19) (Fig. S4), correct integration of the right loxP in four blastocysts (#11, #14, #21, and #27) and nearly correct integration of the right loxP in one blastocyst (#1). No loxP insertion or indel mutation was observed at the left target site in the six blastocysts except for #19, suggesting that the left gRNA has a lower cleavage activity compared with the right gRNA.

### Generation of floxed mice

Based on the results of *Il11* and *Spp1* targeting, we chose microinjection of Cas9 RNP instead of CRISPR-Cas9 vector to generate the founder mice. According to the validation using blastocysts, we increased the concentration of the left gRNA compared with that of the right gRNA to balance the different cleavage activity of these two gRNAs. We microinjected the left and right gRNAs together with the Cas9 protein and ssODN into zygotes generated via the ultra-superovulation method, under several concentration conditions. We then transferred the surviving zygotes to pseudopregnant mice. As summarized in Table 3, higher concentrations of ssODN resulted in a reduction of the birth rate. Tail lysates of all pups were analyzed using the PCRs and two RFLP analyses similar to the blastocyst assay. The results of these assays suggested as follows: five pups, including one pup injected with 2 µM, two with 1.5 µM, and two with 1 µM Cas9, had the left loxP but did not have the right loxP, because they were positive for both the PCR2 and *Psi*I-RFLP analyses, while they were negative for both the PCR3 and *Xmn*I-RFLP analyses (four pups) or for the *Xmn*I-RFLP analysis (one pup). Another five pups, including one injected with 2 µM, two with 1.5 µM, and two with 1 µM Cas9, had the right loxP but did not have the left loxP, because they were positive for both the PCR3 and *Xmn*I-RFLP analyses, while they were negative for both the PCR2 and *Psi*I-RFLP analyses (four pups) or for the *Psi*I-RFLP analysis (one pup). Among 37 pups analyzed, three pups, including two injected with 1.5 µM and one pup with 1 µM Cas9, were positive for all analyses, which were identified as potential floxed mice (Table 3). The PCR products from these pups were cloned and analyzed by DNA sequencing. Of these, one pup was identified with a precisely floxed allele, and the other two pups carried nearly correct integrations (Fig. 4B).

**Table 3.**
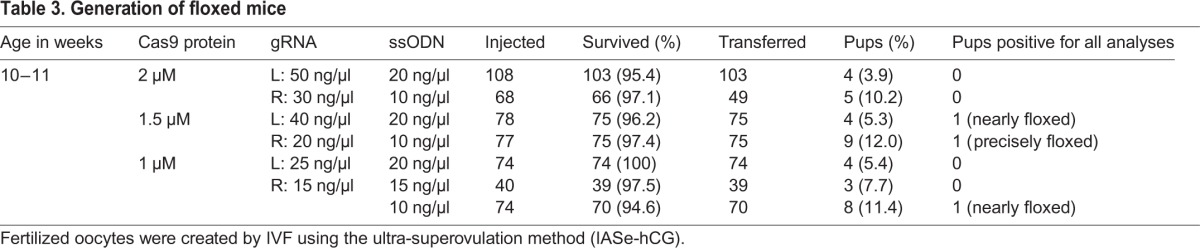
**Generation of floxed mice**

## DISCUSSION

We have previously shown that reproductive engineering techniques were useful for creating knockout mice using a genome-editing technique (Nakagawa et al., 2014, 2015). In this study, we showed that approximately two and three times the number of fertilized oocytes could be generated from sexually matured and immature C57BL/6 female mice, respectively, by IVF using our ultra-superovulation method compared with a conventional superovulation method, consistent with a previous report (Takeo and Nakagata, 2015). To clarify its applicability in the generation of genome-edited mice, we demonstrated the successful production of gene-disrupted, single-amino-acid-substituted, and floxed mice by microinjecting the CRISPR-Cas9 plasmid vectors or RNPs with or without ssODN donors.

We initially aimed to generate various genome-edited mice by microinjection of an all-in-one CRISPR-Cas9 plasmid vector in the C57BL/6 strain because of its simple handling. However, the birth rates of Cas9 nuclease plasmid-injected zygotes were low, similar to other reports using inbred strains (Li et al., 2013; Horii et al., 2014), when compared with B6D2F1 hybrid mice (Mashiko et al., 2013). To solve this problem, we demonstrated that the use of Cas9 protein and synthesized gRNA boosted the birth rate, leading to the successful generation of various genome-edited mice.

Recent achievements using a long double-stranded plasmid donor together with CRISPR-Cas9 enabled the one-step generation of floxed mice (Lee and Lloyd, 2014). In our study, a long ssODN was used instead of the plasmid donor to avoid the labor of donor construction and lower the risk of random integration, and the successful generation of the intended mouse was demonstrated. Unfortunately, three pups containing precise or slightly mutated floxed alleles died within about 2 weeks after birth. This might be explained because they were mosaic founders containing the conditional allele with some other alleles, such as alleles harboring short indel mutations and exon-skipped alleles, which might be disruptive in *Gpcpd1* functions. To overcome this, the knock-in efficiency of ssODN must be further increased by using chemically modified ssODN (Renaud et al., 2016) and/or HDR-enhancing molecules such as Scr7 (Maruyama et al., 2015; Chu et al., 2015) and RS-1 (Song et al., 2016), or the two-step integration of ssODNs should be performed. Another possible reason for the lethality is abnormal pre-mRNA splicing caused by the loxP insertions in the proximity of the target exon. This problem is difficult to solve using chemically synthesized ssODN as a donor, but several methods to synthesize longer single-stranded DNA (ssDNA) were reported recently (Miura et al., 2015; Yoshimi et al., 2016). By incorporating these methods into our strategy, the length limit of chemically synthesized ssODN should be resolved. In summary, our reproductive engineering techniques, especially the ultra-superovulation method, and the improved methods of genome editing will help improve animal welfare and work efficiency when producing GM mice.

## MATERIALS AND METHODS

### Plasmid construction, gRNA synthesis, and preparation of Cas9 protein and ssODN

All-in-one CRISPR-Cas9 plasmids for the *Il11* gene were constructed as previously described (Nakagawa et al., 2015). All-in-one CRISPR-Cas9 plasmids for *Spp1* and *Gpcpd1* genes were constructed using the Multiplex CRISPR/Cas9 Assembly System Kit (Addgene, Cambridge, MA, USA, Kit #1000000055) according to a previous report (Sakuma et al., 2014). Briefly, sense and antisense oligonucleotides for the gRNA templates were annealed and inserted into the pX330-based vectors contained in the kit. Subsequently, gRNA cassettes were tandemly assembled using the Golden Gate assembly method. *In vitro* transcribed gRNAs were prepared according to a previous report (Aida et al., 2015). Briefly, template DNA fragments were generated using PCR amplification from the CRISPR-Cas9 vectors described above using primers containing a T7 promoter sequence. Subsequently, the gRNAs were synthesized using a MEGAshortscript T7 Kit (Life Technologies, Carlsbad, CA, USA), and then purified with a MEGAclear Kit (Life Technologies). The ssODNs were synthesized by IDT (Coralville, IA, USA). The sequences of oligonucleotides for gRNA templates, primers, and ssODNs are listed in Table S3. The recombinant Cas9 protein was obtained from New England Biolabs Japan (Cas9 Nuclease NLS, *Streptococcus pyogenes*; Tokyo, Japan).

### Animals

C57BL/6JJcl strain was purchased from CLEA Japan (Tokyo, Japan). After breeding, C57BL/6J female mice were used as oocyte donors at 4–5 or 10–13 weeks of age. C57BL/6J male mice were used as sperm donors over 12 weeks of age for IVF. ICR mice were used as recipients of injected zygotes at 8–16 weeks of age. All animals were housed under a 12 h dark-light cycle (light from 07:00 to 19:00) at 22±1°C with *ad libitum* food and water. All animal experiments were approved by the Animal Care and Experimentation Committee of the Center for Animal Resources and Development, Kumamoto University, and were carried out in accordance with the approved guidelines.

### IVF and freeze-thawing of fertilized oocytes

The IVF and freeze-thawing procedures were described previously (Nakagawa et al., 2015). Cauda epididymides were obtained from C57BL/6 male mice over 12 weeks of age, and used for IVF as sperm donors. C57BL/6 female mice were intraperitoneally administrated with IASe (0.1 ml IAS and 3.75 IU eCG, CARD HyperOva^®^; Kyudo, Saga, Japan), and then hCG (7.5 IU, Gonatropin; Aska Pharmaceutical, Tokyo, Japan) was intraperitoneally administered to the mice after 48 h (Takeo and Nakagata, 2015). For IASe, pregnant mare serum gonadotropin (PMSG) was used as eCG (Serotropin; Aska Pharmaceutical). They were used as oocyte donors for IVF at 4–5 or 10–13 weeks of age. The generated fertilized oocytes were cryopreserved by a simple vitrification method (Nakagata et al., 2013; Nakao et al., 1997). At later time points, the cryopreserved oocytes were thawed and used for microinjection and transfer.

### Microinjection

Each CRISPR-Cas9-plasmid DNA was diluted in DNase-free PBS, and then injected into the pronucleus of fertilized oocytes. For the microinjection of RNPs, *in vitro* transcribed gRNA(s) and Cas9 protein were mixed with or without ssODN in 0.1 TE buffer (Aida et al., 2015). The concentration of plasmid DNA, ssODN, and RNP is described in Tables 1, 2, 3 and Table S2, except that 5 ng/µl plasmid DNA and 10 ng/µl ssODN was injected for single blastocyst assay to validate the generation of the floxed mice. The injected oocytes were cultured in potassium simplex optimized medium with amino acids (KSOM-AA), prepared as previously described (Lawitts and Biggers, 1993; Ho et al., 1995), at 37°C in 5% CO_2_ and 95% humidified air for about 1 h. Surviving oocytes were transferred to the oviducts of pseudopregnant ICR female mice.

### Single blastocyst assay

The single blastocyst assay was performed according to a previous report (Nakagawa et al., 2015). To detect the targeted *Spp1* gene, PCR was performed using KOD FX (Toyobo, Osaka, Japan) with the Spp1 F and R primers listed in Table S3 under the following conditions: 95°C for 1 min, followed by 38 cycles of 98°C for 10 s, 60°C for 30 s, and 68°C for 30 s. Each PCR product was subjected to automatic electrophoresis using MultiNA (Shimadzu Corporation, Kyoto, Japan). The PCR products were purified and analyzed by RFLP analyses using *Bsm*FI (New England Biolabs Japan), then the PCR products identified as positive were analyzed by direct sequencing using an ABI 3130 Genetic Analyzer (Life Technologies) with a BigDye Terminator v1.1 Cycle Sequencing Kit (Life Technologies). To detect the targeted *Gpcpd1* gene, PCR1 was performed with the F and R primers listed in Table S3 under the following conditions: 95°C for 1 min, followed by 39 cycles of 98°C for 10 s, 61°C for 30 s, and 68°C for 45 s. PCR2 or PCR3 were performed with the F and L-lox primers or R-lox and R primers listed in Table S3 under the following conditions: 95°C for 1 min, followed by 39 cycles of 98°C for 10 s, 57°C for 30 s, and 68°C for 25 s. Each PCR product was subjected to automatic electrophoresis using MultiNA. The PCR1 products were purified and analyzed by RFLP analyses using *Psi*I (New England Biolabs Japan) or *Xmn*I (New England Biolabs Japan), then the PCR1 products identified as positive in all analyses were analyzed by direct sequencing.

### Analysis of pups

Tail lysates of pups were prepared by an alkaline lysis method and PCR was performed using KOD FX with each primer sets. For the analysis of *Il11* mutants, the IL11 F and R primers listed in Table S3 were used according to a previous report (Nakagawa et al., 2015). Each PCR product was analyzed by direct sequencing. For the analysis of *Spp1* gene, PCR were carried out with 37 cycles, and then the products were analyzed as described in the same way as the single blastocyst assay. For the analysis of *Gpcpd1* gene, three kinds of PCR were carried out with 37 cycles, and then the products were analyzed as described in the same way as the single blastocyst assay. The PCR1 products harboring floxed mutations were subcloned into a pTA2 vector using Target Clone -Plus- (Toyobo). Each subcloned vector was analyzed by sequencing.
